# Supplementary material for: Genome evolution and divergence in cis-regulatory architecture is associated with condition-responsive development in horned dung beetles
Source: PLoS Genet. 2024 Mar 5;20(3):e1011165. doi: 10.1371/journal.pgen.1011165 (PMC10942260; doi:10.1371/journal.pgen.1011165)

**Supplementary Figure 1:** Comparison of lineage-specific and shared OCRs repetitive element content. **A)** Percent of total OCRs containing at least one repetitive element. **B)** Number and class of repetitive element for the 100 most abundant repeats present in each OCR type.


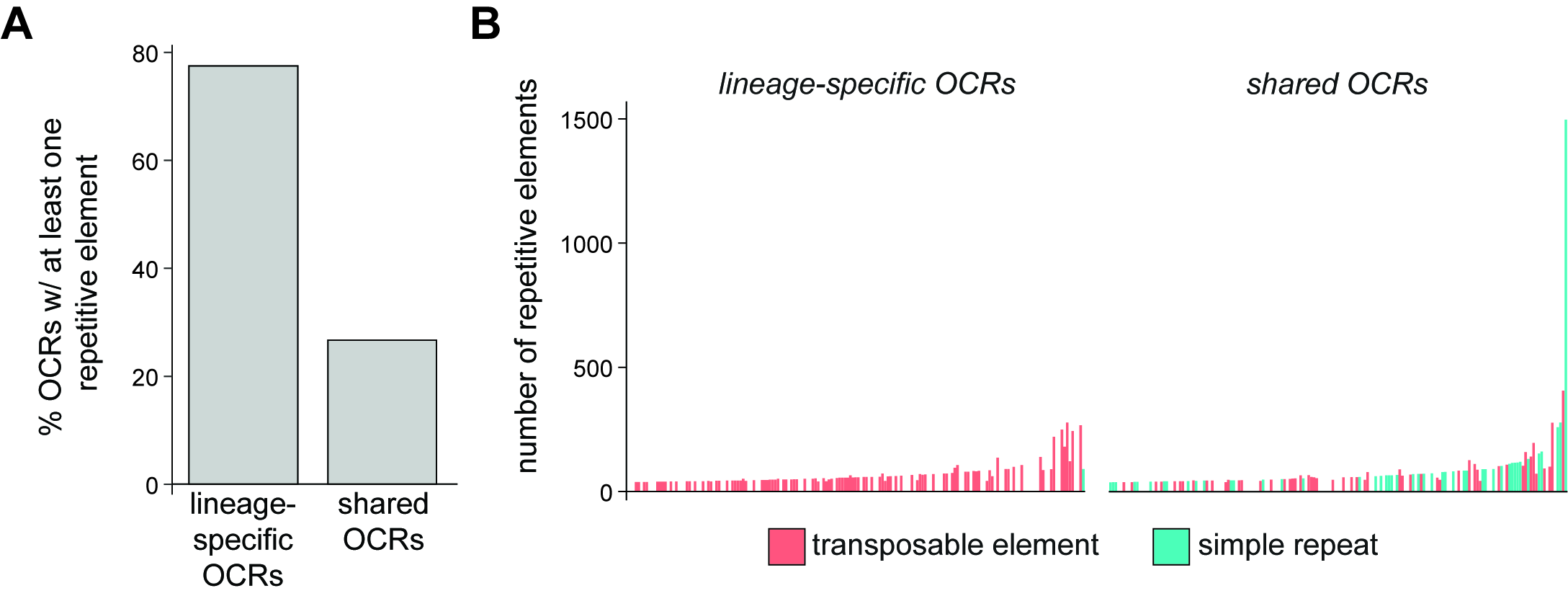

Supplement: S1 Fig — A) Percent of total OCRs containing at least one repetitive element. B) Number and class of repetitive element for the 100 most abundant repeats present in each OCR type. (DOCX) [file pgen.1011165.s006.docx]
