# Supplementary material for: Genome evolution and divergence in cis-regulatory architecture is associated with condition-responsive development in horned dung beetles
Source: PLoS Genet. 2024 Mar 5;20(3):e1011165. doi: 10.1371/journal.pgen.1011165 (PMC10942260; doi:10.1371/journal.pgen.1011165)
Supplement: Attachment — Submitted filename: PGenetic-D-23-01079_Review.pdf [file pgen.1011165.s008.pdf]

Genome evolution and divergence in cis-regulatory architecture underlie condition responsive development in horned dung beetles.

Phillip L. Davidson, Armin P. Moczek

Suitable Quality?: Yes
Sufficient General Interest?: Yes
Conclusions Justified?: Yes
Clearly Written?: Yes
Procedures Described?: Yes
Supplemental Material Warranted?: Yes

Dr. Davidson and his colleague presented an interesting study on the genome evolution and divergence of cis-regulatory elements in horned dung beetles in relation to phenotypic plasticity and sexual dimorphism. The authors sequenced and assembled chromosomal-level genomes for three species of dung beetles: *Onthophagus taurus*, *Onthophagus sagittarius*, and *Digitonthophagus gazella*. These genomes exhibited high contiguity and BUSCO scores, suggesting their accuracy as reference genomes. In their comparison of genome characteristics across the three dung beetle species, the authors revealed variations in genome size and found in *O. sagittarius*, the expansion of non-coding repetitive elements is to be a significant contributor to genome size variation, whereas in D. gazella, the acquisition of species-specific genes played a crucial role in genome expansion.

The authors then shifted their focus to the evolution of coding sequences in dung beetles, identifying various gene families that have rapidly evolved in these insects, potentially linked to their unique adaptations. These candidate genes are associated with metabolism, regulation of metamorphosis, odorant receptors.

Next, the authors carried out ATAC-seq on O. taurus low and high nutrition fed male, and females head horns. The authors find a substantial difference in Open Chromatin Regions (OCR) accessibility between males reared in high vs. low nutrition conditions and not many OCR differences between females in nutrition conditions. As this would be expected, as male shows phenotypic plasticity of horn between two nutrition and female lacks horn in both cases. The study also highlights an intriguing finding: a substantial number of OCRs more accessible in minor males are located on the X chromosome, which implies that genes on the X chromosome may play a crucial role in regulating the plasticity of horn development. The study also shows that these recently evolved CREs are more prevalent in nutrition-responsive horn development compared to sex-responsive horn development, which more likely uses ancestral CREs.

Overall, I found the study to be well executed. The introduction was elaborate. The inclusion of a new genome assemblies is always important. The author also shows how comparative genome analysis can be utilized in better insight into lineage specific evolution. Comparative chromatin analysis between different nutrition conditions is interesting. It’s one of the first study I could think of to perform chromatin assays in beetle. I only have few minor comments.

- Line 167-179: The authors discuss the genome size expansion observed in these dung beetle species: non-coding repetitive elements in *O. sagittarius* and coding sequence expansion in D. gazella. It would be nice, if authors could explain a bit more on what could be the possible explanation for D. gazella genome expansion. If the coding sequence expansion in *D. gazella* also has to do with any repetitive elements or not, particularly?

- In Figure 4A, a Venn diagram illustrates the presence of shared and uniquely differentially accessible head horn OCRs as identified in each pairwise comparison of *O. taurus* individuals. Are the shared differential peaks observed in the two pairwise comparisons are OCRs present in the same tissue in those comparisons? For example, whether the 122 differential peaks shared between major vs. minor males and major males vs. females are OCR to only major male tissue. If this is not the case, it would be nice, if the authors could provide bit more information on any specific peaks in only major males, minor males, and females that are OCRs in the respective pairwise comparisons.

  In Figure 4B, and in lines 395 to 409, the authors discuss the enrichment of different transcription factor motifs in OCRs in the context of nutrition and sex-based comparisons. Are any of these TF motifs enriched in nutrition or sex-based comparisons associated with pioneer factors? The differences in the presence of many non-overlapping peaks between the two sets could be explained if there are specific pioneer factors involved in plasticity and sexual dimorphism.

- Line 427-433. Authors note that 32.9% of the nutrition-responsive OCRs in male head horn development are comprised of recently evolved regions, for which there are no matching sequences found in other genomes. Like I mentioned earlier, authors may need to investigate whether these OCRs, unique to the O. taurus lineage, contain any distinct transposable elements (TE). Transposable elements are known to significantly influence the evolution of lineage-specific regulatory elements. It would be intriguing to examine whether the evolution of head horn plasticity is associated with specific TE elements.
