# Supplementary material for: Genome evolution and divergence in cis-regulatory architecture is associated with condition-responsive development in horned dung beetles
Source: PLoS Genet. 2024 Mar 5;20(3):e1011165. doi: 10.1371/journal.pgen.1011165 (PMC10942260; doi:10.1371/journal.pgen.1011165)
Supplement: Attachment — Submitted filename: DavidsonEtAl_response_to_reviewers.docx [file pgen.1011165.s009.docx]

**Response to Reviewers**

We thank the reviewers for their time and very helpful comments on the manuscript, which we feel has improved the content, clarity, and interpretation of this work. Below in blue text, we describe has each comment has been addressed in this revised submission, providing line numbers where appropriate to denote where these changes reside in the revised manuscript.

**Reviewer 1**

This is an interesting and well-presented manuscript investigating the genetic/genomic basis of a novel trait that is affected by both sex and nutrition relation polymorphism. The manuscript presents the genomes of three species of dung beetle, assembly and annotation data associated, extending to an evolutionary analysis of gene presence/absence. The authors then present ATAC-seq data to indicate chromatin availability, allowing them to investigate the regulatory landscape of these traits, and, through clever use of different species, dissect the influence of sex and diet on the growth of beetle horns.

In my opinion this is an excellent manuscript well deserving of publication in PLOS genetics. It is a careful analysis of the regulation of a novel trait- and thus adds a great deal to our understanding of how such traits evolve, and how they are environmentally regulated. This more comprehensive approach indicates the importance of cis-regulatory element evolution in beetle horns, and the authors final comments in the conclusions are a very important challenge to others in this field.

I have a few, probably, minor issues that I think the authors need to address before publication.

1) The first is the data on gene numbers in these species. The authors present three very good genomes of Dung beetles. The finding that one of these species has considerably more gene predicted than the others, or indeed other beetle genomes, is a surprise. If this is truly the case I hope the authors are following up on this work. In most of the rest of the analyses I don’t see how these extra genes are included and one might suggest that our tools for analysing gene loss gain etc in these situations are poor- so perhaps the importance of these genes is down played. It would be nice to see a paragraph or two discussing these genes.

Thank you for this suggestion and a similar comment regarding the origin of these extra gene models in *D. gazella* was also raised by Reviewer 2 (see their 1^st^ comment). For consistency, we have supplied identical responses to both comments. Upon revisiting these gene annotations, we noticed that most of these extra gene models that appeared to be species-specific were located on non-chromosomal length scaffolds in the assembly and after further investigation, were determined to be prokaryotic contamination. In this revised submission, we have removed these contaminants from consideration, bringing the gene content of *D. gazella* to a more reasonable 18,889 genes. Figures 1C and 2B,C have been revised accordingly, along with the text on lines 166, 168-173. In short, the number of gene models and genome size of *D. gazella* are within the expected range and we no longer highlight any sort of genome expansion of this species. We apologize for not catching this artefact earlier and are thankful we can correct this issue at this point.

It is also a serious possibility that these novel genes are a result of mis-annotation. Clearly annotations improve with high quality RNAseq data being added- and this is indicated in the methods as having happened. The reference however is to a paper with no RNAseq datasets in it- So I am unsure as to what datasets are being used- is it possible that imbalances in the RNAseq data sets or amount of RNAseq used in each genome annotation have led to the appearance of the extra genes? Can the authors please indicate where their RNAseq comes from, and perhaps provide a table so we can be clear what RNAseq data has gone into each annotation process.

Apologies for the citation typo and resulting confusion. In the revised manuscript, we have corrected the RNA-seq citation to Casasa et al. 2020 (Casasa, S., Zattara, E.E., and Moczek, A.P. (2020). Nutrition-responsive gene expression and the developmental evolution of insect polyphenism. Nat Ecol Evol 4, 970–978. 10.1038/s41559-020-1202-x.), which includes RNA-sequencing data from all three species. Since all of the RNA-sequencing data came from a single study we feel a separate table is not necessary in this case.

2) The authors present BUSCO results as the key quality measure for their genomes. This is OK, though it would be nice to see others if available. BUSCO however comes in lots of different forms- the Authors don’t state what version of BUSCO they used, nor what dataset they tested against. This is important as it is relative easy to achieve 95% BUSCO scores if testing your genome against a eukaryote dataset, harder if it is a beetle one. Could the authors please provide this information?

Thank you for pointing out this lack of detail regarding the BUSCO analysis in the original manuscript. In the original submission, we used BUSCO v.5.4.2 and referenced the broad metazoan lineage dataset (metazoan_odb10). To ensure we did not misrepresent the completeness of these assemblies, in this revised submission we have included results of the same BUSCO analysis against the insect dataset (insecta_odb10) which represents the most specific clade available for BUSCO v5.4.2 for beetles (dipterans, hymenopterans, and lepidopterans were the only order-specific datasets available). Interestingly, testing against this more specific dataset improved the BUSCO score for each assembly by ~1.5% (complete single copy hits) and reduced the duplication scores slightly. We have edited the text (lines 163) and Figure 1 to represent these more accurate BUSCO scores in this revised submission, and now provide details of this analysis in the Methods (lines 595-599).

3) In Figure 4 the authors indicate some key potential transcription factor binding sites in their data- one of these is labelled Bicoid. As I understand it the Bicoid gene is limited to certain groups in the Diptera (see https://doi.org/10.1002/bies.20285). What then is this binding site- is it Hox3 , or Orthodenticle? I assume this has been identified through a Drosophila data set which has brought up this motif and labelled it as it is. The authors should explain this, and if possible, indicate what protein they think might be binding this motif.

Thank you for raising this concern and yes, the reviewer is correct in that Bicoid is not present in the *Onthophagus* genome and the matching motif comes from a *Drosophila* dataset. During revisions, we have conducted a motif similarity search for the “bicoid” hit using Jaspar (<https://jaspar.elixir.no/>) and MEME- TomTom (<https://meme-suite.org/meme/tools/tomtom>) in an effort to identify possible “true” matches for this motif. These analyses identified *dorsal*, *scalloped*, and *pitx1*, among other less significant hits, as possible matches to the enriched motif. Given the ambiguity and need for experimental validation, we now explain this interpretive limitation in the Methods on lines 731-735 but refrain from proposing what the true match may be due to the remaining uncertainty.

4) Given the authors have Hi-C data as well as ATAC-seq (and I know the Hi-C is from different tissues and stages so this may be pointless), but does the Hi C interaction map confirm or provide extra information about the chromatic accessibility data?

Thank you for this great suggestion and we agree the addition of HiC data could help resolve regulatory domains in this system. From the HiC data used in the genome assembly, we were only able to confidently identify 173 topologically associated domains (TADs) (66 at 10Kb, 70 at 25Kb, and 37 at 50Kb resolution) from a single whole animal tissue, which is considerably lower than expected of a formal HiC experiment (several thousand for a cell type). This is likely attributable to low sequencing depth, low library complexity/cell count, and sampling an entire organism instead of a particular tissue type that is required of a formal HiC analysis of TADs. So while our HiC experiment was sufficient for genome assembly, we are very hesitant to apply this data to analyzing TADs in dorsal head epithelia. Still, a formal analysis of the presence (and evolution) of TAD boundaries in this tissue type represents an exciting future direction for understanding condition-specific gene regulation.

5) There are a lot of bioinformatic analyses in this manuscript- and the availability of data is good. What I can’t find is any availability of scripts. For a paper like this, I would hope to see a GitHub (or similar) archive where I could find the scripts to replicate these results. I think this is really important, given the outcomes of this paper, and the need to compare with similar novel or non novel traits. Providing code is the best way to ensure this research is reproducible and widely used.

Thank you for this reminder on providing scripts used for these analyses. In this revised submission, we provide a list of scripts used for completing the major steps of this work (incl. ATAC-seq analysis, gene family evolution analysis, genome annotation/comparison) as a Supplementary Text File and note their availability on Dryad in the Data Availability section.

**Reviewer 2:**

Dr. Davidson and his colleague presented an interesting study on the genome evolution and divergence of cis-regulatory elements in horned dung beetles in relation to phenotypic plasticity and sexual dimorphism. The authors sequenced and assembled chromosomal-level genomes for three species of dung beetles: *Onthophagus taurus*, *Onthophagus sagittarius*, and *Digitonthophagus gazella*. These genomes exhibited high contiguity and BUSCO scores, suggesting their accuracy as reference genomes. In their comparison of genome characteristics across the three dung beetle species, the authors revealed variations in genome size and found in *O. sagittarius*, the expansion of non-coding repetitive elements is to be a significant contributor to genome size variation, whereas in D. gazella, the acquisition of species-specific genes played a crucial role in genome expansion.

The authors then shifted their focus to the evolution of coding sequences in dung beetles, identifying various gene families that have rapidly evolved in these insects, potentially linked to their unique adaptations. These candidate genes are associated with metabolism, regulation of metamorphosis, odorant receptors.

Next, the authors carried out ATAC-seq on O. taurus low and high nutrition fed male, and females head horns. The authors find a substantial difference in Open Chromatin Regions (OCR) accessibility between males reared in high vs. low nutrition conditions and not many OCR differences between females in nutrition conditions. As this would be expected, as male shows phenotypic plasticity of horn between two nutrition and female lacks horn in both cases. The study also highlights an intriguing finding: a substantial number of OCRs more accessible in minor males are located on the X chromosome, which implies that genes on the X chromosome may play a crucial role in regulating the plasticity of horn development. The study also shows that these recently evolved CREs are more prevalent in nutrition-responsive horn development compared to sex-responsive horn development, which more likely uses ancestral CREs.

Overall, I found the study to be well executed. The introduction was elaborate. The inclusion of a new genome assemblies is always important. The author also shows how comparative genome analysis can be utilized in better insight into lineage specific evolution. Comparative chromatin analysis between different nutrition conditions is interesting. It’s one of the first study I could think of to perform chromatin assays in beetle. I only have few minor comments.

- Line 167-179: The authors discuss the genome size expansion observed in these dung beetle species: non-coding repetitive elements in *O. sagittarius* and coding sequence expansion in D. gazella. It would be nice, if authors could explain a bit more on what could be the possible explanation for D. gazella genome expansion. If the coding sequence expansion in *D. gazella* also has to do with any repetitive elements or not, particularly?

Thank you for this suggestion and a similar comment regarding the origin of these extra gene models in *D. gazella* was also raised by Reviewer 1 (see their 1^st^ comment). For consistency, we have supplied identical responses to both comments. Upon revisiting these gene annotations, we noticed that most of these extra gene models that appeared to be species-specific were located on non-chromosomal length scaffolds in the assembly and after further investigation, were determined to be prokaryotic contamination. In this revised submission, we have removed these contaminants from consideration, bringing the gene content of *D. gazella* to more reasonable 18,889 genes. Figures 1C and 2B,C have been revised accordingly, along with the text on lines 166, 168-173. In short, the number of gene models and genome size of *D. gazella* are within the expected range and we no longer highlight any sort of genome expansion of this species. We apologize for not catching this artefact earlier and are thankful we can correct this issue at this point.

- In Figure 4A, a Venn diagram illustrates the presence of shared and uniquely differentially accessible head horn OCRs as identified in each pairwise comparison of *O. taurus* individuals. Are the shared differential peaks observed in the two pairwise comparisons are OCRs present in the same tissue in those comparisons? For example, whether the 122 differential peaks shared between major vs. minor males and major males vs. females are OCR to only major male tissue. If this is not the case, it would be nice, if the authors could provide bit more information on any specific peaks in only major males, minor males, and females that are OCRs in the respective pairwise comparisons.

We agree this is a great point for clarification. Interestingly, the OCRs that are differentially accessible in our pairwise comparisons are present in both tissues being analyzed (i.e. the 122 differentially accessible peaks in major males are present in females and minor males as well, just consistently differentially accessible in major males). We have added the phrase “*OCRs are detected in each tissue type*” in the legend of Figure 4A to clarify this point to readers.

- In Figure 4B, and in lines 395 to 409, the authors discuss the enrichment of different transcription factor motifs in OCRs in the context of nutrition and sex-based comparisons. Are any of these TF motifs enriched in nutrition or sex-based comparisons associated with pioneer factors? The differences in the presence of many non-overlapping peaks between the two sets could be explained if there are specific pioneer factors involved in plasticity and sexual dimorphism.

Thank you for this helpful comment. With this revision, we have checked our lists of enriched TF binding sites for potential upstream chromatin regulatory molecules such as pioneer factors. While we did not detect an enrichment of any pioneer binding sites in our differentially accessible OCRs, we agree this is an important next step to identifying how these tissue-specific differences in chromatin may arise in the first place. Accordingly, in our revised manuscript, we have added the following statement of future direction on lines 487-490: “*Future work aimed at identifying which upstream regulatory molecules (e.g. pioneer factors) generate condition-dependent differences in chromatin accessibility represents will be critical to more mechanistic understanding of the evolution of head horn regulation in this species.”*

- Line 427-433. Authors note that 32.9% of the nutrition-responsive OCRs in male head horn development are comprised of recently evolved regions, for which there are no matching sequences found in other genomes. Like I mentioned earlier, authors may need to investigate whether these OCRs, unique to the O. taurus lineage, contain any distinct transposable elements (TE). Transposable elements are known to significantly influence the evolution of lineage-specific regulatory elements. It would be intriguing to examine whether the evolution of head horn plasticity is associated with specific TE elements.

Thank you for this great comment. In an effort to understand how these unique OCRs may have originated, in this revised manuscript we now compare TE content of unique vs. non-unique OCRs in *O. taurus*. Interestingly, we note that a much greater proportion of unique OCRs contain at least one repetitive element (77.5%) compared to non-unique OCRs (26.7%). Further, repetitive element content of unique OCRs is dominated by transposable elements whereas non-unique OCRs include both TEs and simple repeats, further suggesting TEs may have played an outsized role in the evolution of these unique OCRs. We now include a Supplementary Figure illustrating these results and make a brief, but cautious, note in the revised text on lines 515-518.

**Reviewer 3:**

This study examines the genetic basis of adaptation in dung beetles using a combination of comparative genomics and experimental genomic work on regulatory variation associated with evolution of nutritional plasticity of an ornament (horn size). To do so the authors generate three new chromosome level assemblies from different dung beetles (combining PacBio HiFi and Hi-C technology) and combine these genomes with already existing assemblies from other beetle species to examine the genome evolution (losses/gains of genes and selection on them) that might be associated with dung beetle specific adaptations. The main findings from this was the identification of 200-300 evolving orthogroups and around 100 losses from the dung beetle node. These rapidly involving orthogroups in dung beetle node include some interesting gene families such as the juvenile hormone expansion in O.taurus and the large variation in TOR copy number between O.taurus and O.sagittarius.

Indeed, the gene expansion of the TOR gene is very interesting both from the point of view of its known growth regulatory role but also from some recent research in seed beetles showing that the Y chromosome harbours copy number variation in TOR that contributes to differences in body size between different male Y haplotypes (Kaufmann et al 2023 MBE*).

The other part of the ms examines the chromatin landscape across different comparisons (horn size differences and sex differences) using ATAC seq to better understand the CREs involved in nutrition induced morphological differences. These data come from the epithelial cells of the head where males develop horns, an important consideration in the experimental design which makes interpretation and link to horn morphology more robust. The main finds here are the identification of both nutrition induced changes in CRE accessibility in males and differences between the sexes which led to the identification of some novel candidate genes that can be tested in a functional setting. Combining the OCR analysis with differential expression also seem to, at least partly, establish a correlation between the two for candidate genes identified (although not at the genome wide level) which to my knowledge is rarely done thus far. Enrichment of DA OCRs on the X chromosome for the large honred vs small horned and large horned vs female comparisons is interesting and also a bit surprising…

Overall I think this ms explores a lot of interesting questions surrounding the role of plasticity and its genetic basis and the design of the experiment and the interpretations seem largely sound to me (though see comments below).

Major issues:

- While the gene family expansion analysis across the different species are interesting I think at times too much is made about their involvement in nutritional plasticity. There are surely many differences between the compared species and so I do not think that, for example, the TOR finding can be attributable to species differences in evolutionary exaggeration and secondary loss of male polyphenism (see lines 233-236 and 254-257 for examples where this is claimed). Differences in body size between species could potentially also be linked to TOR copy number variation (Kaufmann et al 2023), just to give one alternative interpretation. I would therefore advise to be less speculative in how these findings are discussed and interpreted not to fall into adaptive storytelling. It is good this is brought up specifically on lines 245-248 but perhaps it should be emphasized throughout since also the species comparison between taurus and sagittarius is set up as identifying gene families involved in nutritional plasticity but there are surely many other species differences between them apart from nutritional plasticity.

We thank the reviewer for this comment and agree our suggestion of causality may have been too strong in this section. In the revised manuscript, we have made several changes to reflect alternative explanation for differences in gene copy number that may or may not be adaptive to temper our interpretation of these copy number differences. Please see:

lines 236-238: “While gene family size changes and non-synonymous mutations are not necessarily indicative of adaptation, these analyses provide predictions of candidate genes and pathways for further assessment.”

lines 278: “copy number changes or adaptations,”

lines 310-311: “Given the correlative nature of this result and the fact that TOR copy number can vary widely among lineages,”

In addition to the comment in the original submission on lines 295-299: “However, like all gene family evolution analyses, associations of orthogroup expansions with dung beetle adaptations are speculative, and functional assessment of these genes and pathways will be necessary to validate the predictions mentioned above.”

- A FDR of 10% for calling differential accessibility is rather lenient (p617-619)… I think it would be useful to report at 5% level what you find as well and keep a table in the Supp mat for this. And using a FDR of 10% just further highlight the importance of interpreting these results with caution.

Thank you for this helpful point. A 10% FDR was chosen given the exploratory nature of this study and inherent noise of ATAC-seq data, however the point is well-taken that a 5% FDR is more typical functional genomic studies. In response, we have edited Figure 3 to include a 5% cutoff line for differential accessibility and provide the number of significant OCRs in each panel below the 10% and 5% cutoff. The figure legend has also been edited accordingly. While the absolute number of significant OCRs changes, importantly, the patterns of differential accessibility remain the same. The FDR of each comparison is provided in Supplementary Data 4, so readers will have access to OCRs with higher and lower support thresholds.

Minor:

- I think the title is too conclusive in claiming that the observed changes at both the genomic level and regulatory level underlie the condition dependent development. It should be revised to reflect the fact that these are after all correlative analyses

Thank you for this suggestion. We have changed the word “underlie” to “is associated with” so as not to imply a causal relationship between our findings.

- line 274: do you mean the horns or other developmental phenotypes?

Thank you for pointing out this ambiguity. In the revised manuscript, we have changed this phrase from “some of which may play adaptive roles in these species’ derived developmental phenotypes.” to “some of which may play adaptive roles in these species’ development.” so as not to sound too conclusive as to how these mutations may affect function.

- I find Fig 2D quite difficult to interpret but dont have any suggestions on how it could be improved…

We agree that Fig 2D is complex, in particular because the color coding corresponds to branches within the dung beetle/onthophagine phylogenetic tree, so the reader has to examine figure 2D in relation to fig 2A. However, all elements in Fig 2 are designed this way, that is once the color coding in the phylogeny is established all subsequent data presented follow the same referencing scheme. Further, we did try alternate forms of presenting these results but never managed to arrive at something we found more satisfying or comprehensive. After discussing this further we in the end decided not to change fig 2D and keep the original design.

- Fig 3: is the GW significant line the FDR at 10% line? Using Bonferroni correction it should be -log10(0.05/68000) = 6.1, which would leave a significantly smaller amount of DA OCRs.

Apologies for this error and thank you for catching this. Rather than log10, the y-axis of this figure was generated with the natural log. The axis label should now make sense, as -ln(0.1) = ~2.3.
