# Supplementary material for: Genome evolution and divergence in cis-regulatory architecture is associated with condition-responsive development in horned dung beetles
Source: PLoS Genet. 2024 Mar 5;20(3):e1011165. doi: 10.1371/journal.pgen.1011165 (PMC10942260; doi:10.1371/journal.pgen.1011165)
Supplement: Attachment — Submitted filename: Review_R1.docx [file pgen.1011165.s010.docx]

Genome evolution and divergence in cis-regulatory architecture underlie condition responsive development in horned dung beetles.

Phillip L. Davidson, Armin P. Moczek

Suitable Quality?: Yes 
Sufficient General Interest?: Yes 
Conclusions Justified?: Yes 
Clearly Written?: Yes 
Procedures Described?: Yes 
Supplemental Material Warranted?: Yes 

The authors have done their best to address my critiques. I have no more comments on the revised version. I do believe the reworked manuscript is of high quality and should be published. 
